# Development and application of an antibody detection ELISA for *Haemophilus parasuis* based on a monomeric autotransporter passenger domain

**DOI:** 10.1186/s12917-019-2128-x

**Published:** 2019-12-03

**Authors:** Yunbao Liu, Yujiao Du, Yuping Song, Yang Tian, Yi Qi, Qinxue Zhang, Qigai He, Xiangru Wang, Huanchun Chen, Xuwang Cai, Xiaojuan Xu

**Affiliations:** 1 State Key Laboratory of Agricultural Microbiology, College of Veterinary Medicine, Huazhong Agricultural University, Wuhan, Hubei China; 2 The cooperative Innovation Center for Sustainable Pig Production, Key Laboratory of Preventive Veterinary Medicine in Hubei Province, Wuhan, 430070 Hubei China

**Keywords:** *Haemophilus parasuis*, ELISA, Antibody detection, Autotransporter

## Abstract

**Background:**

*Haemophilus parasuis* is a commensal pathogen in the swine upper respiratory tract and causes Glässer’s disease. Surveillance, screening for infection, and vaccination response of *H. parasuis* is hindered by the lack of a rapid antibody detection method.

**Results:**

In the present study, a monomeric autotransporter was identified as a novel antigen for developing an indirect ELISA. The autotransporter passenger domain (Apd) was expressed, purified, and demonstrated to be specific in ELISA and western blotting. Mouse antiserum of recombinant Apd (rApd) recognized native Apd in the 15 serotype reference strains and five non-typeable isolate stains, but showed no reaction with seven other bacterial pathogens. The rApd ELISA was optimized and validated using 67 serum samples with known background, including 27 positive sera from experimentally infected and vaccinated pigs along with 40 negative sera that had been screened with *H. parasuis* whole cell ELISA from clinically healthy herds. The rApd ELISA provided positive and negative percent agreements of 96.4 and 94.9%, respectively, and an AUC value of 0.961, indicating that the assay produced accurate results.

**Conclusion:**

Apd was a universal antigen component among 15 serotype and non-typeable strains of *H. parasuis* and was also specific to this pathogen. The rApd ELISA could detect antibodies elicited by *H. parasuis* infection and vaccination, thereby exhibiting the potential to be applied for Glässer’s disease diagnosis, *H. parasuis* vaccination evaluation, and large-scale serological surveillance.

## Background

*Haemophilus parasuis,* a member of the family *Pasteurellaceae*, constitutes an early colonizer of the swine upper respiratory tract [1]*. H. parasuis* causes swine Glässer’s disease, which is characterized by fibrinous polyserositis, polyarthritis, and meningitis [2]. Glässer’s disease is distributed worldwide, affecting 1–4 month-old pigs especially under conditions of stress [3]. Conversely, *H. parasuis* does not result in systemic infection in healthy herds despite commonly colonizing the swine upper respiratory tract; thus, isolation from the nasal cavity is not used for diagnosis of Glässer’s disease [4, 5]. Rather, its isolation such as through bacterial culture from the systemic tissue sites of pigs showing clinical signs and pathological lesions represents the gold standard for diagnosis [1, 3]. This method, however, is complicated and time-consuming. In comparison, serological tests are simpler and faster. Moreover, Glässer’s disease tends to occur following infection by porcine reproductive and respiratory syndrome or other bacterial pathogens [6]. In such cases, it is also necessary to specifically diagnose Glässer’s disease according to serological assays. Serological assays are further required for epidemiological surveillance of Glässer’s disease as well as for evaluating the antibodies elicited by the several commercial inactive vaccines of *H. parasuis*.

To date, complement fixation assays, indirect hemagglutination assays (IHA), and enzyme-linked immunosorbent assays (ELISA) have been reported for *H. parasuis* antibody detection [5, 7–10]. However, IHA utilizing the supernatants of sonicated or boiled bacteria as antigens, and ELISA incorporating the supernatants of boiled bacteria or dialyzed hot phenol water extracts of the bacteria (polysaccharides or lipopolysaccharides) as antigens, elicited unstable or negative results [8]. Alternatively, IHA and ELISA based on oligopeptide permease A (OppA) could discriminate the sera of the convalescent pigs from those of specific pathogen-free and clinically normal healthy pigs [4, 5, 10]. Nevertheless, OppA protein is also present in other swine bacterial pathogens, such as *Actinobacillus pleuropneumoniae*, *Escherichia coli, Salmonella enteric and Yersinia pestis* [5, 11 12], which might afford cross-reactions because of similar antigen epitopes.

Compared with complement fixation assay and IHA, ELISA is commonly applied and thus is considered to represent the preferred method. However, the positive and negative percent agreement (sensitivity and specificity) of ELISA is affected by several factors including, respectively, the localization and immunogenicity of the antigen [13], and its similarity to that of other related organisms. Additionally, the classification of an antigen as type- or species-specific will define the range of the detected strains [14]. Therefore, antigen choice is crucial for the development of an antibody detection ELISA.

Autotransporters consist of a cleavable N-terminal signal peptide, a functional passenger domain exposed to the outer membrane or released into the external environment, and a C-terminal region involved in the formation of a transmembrane pore [15]. In the present study, three autotransporter passenger domains of *H. parasuis* were determined to represent candidate antigens for *H. parasuis* indirect ELISA, with a monomeric autotransporter passenger domain (Apd) being selected as the optimal antigen. An ELISA antibody detection assay based on the rApd was consequently developed, evaluated, and applied.

## Results

### Expression and screening of rEspP1, rEspP2, and rApd

**Fig. 1:**
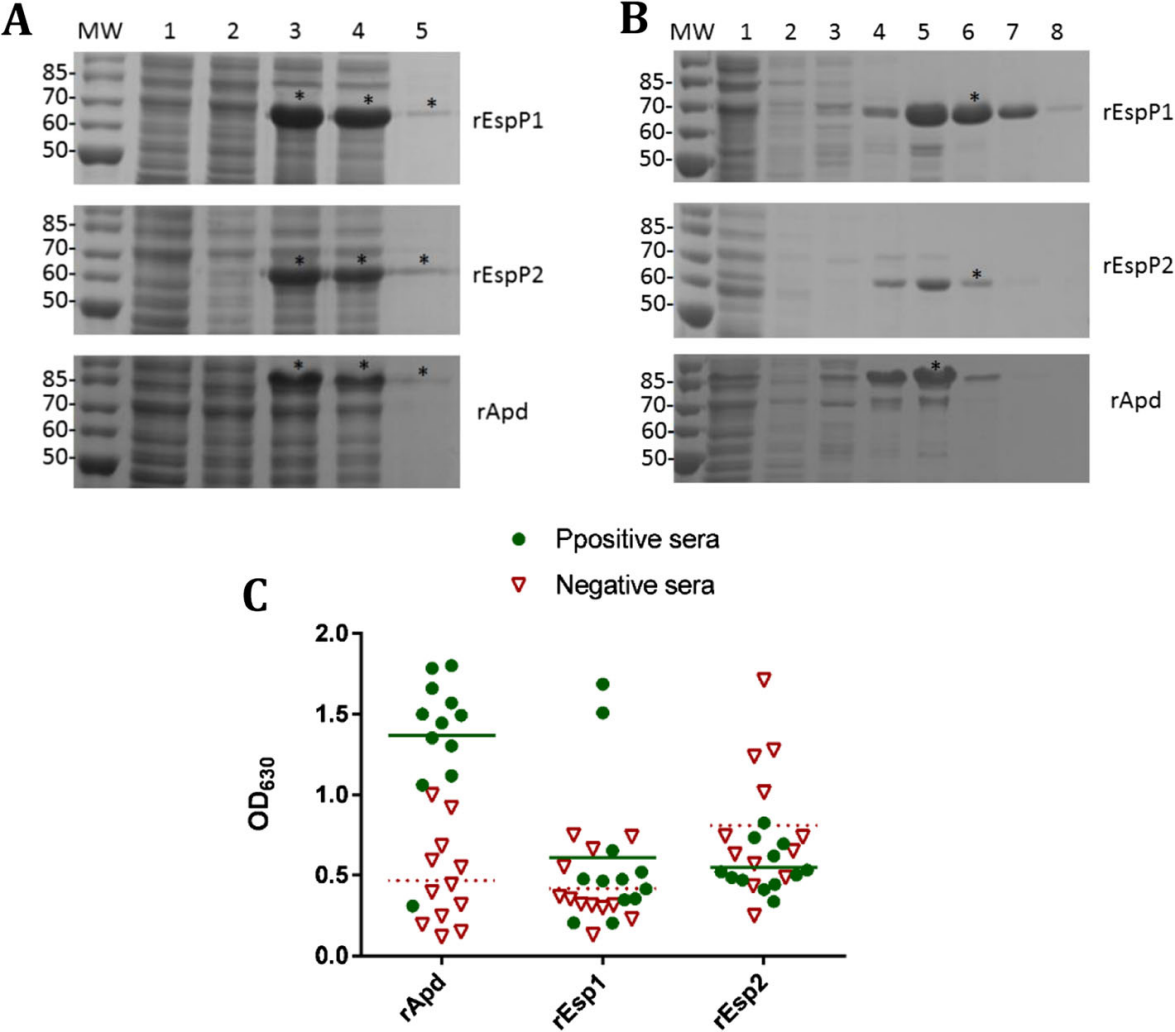
Expression, purification, and screening of EspP1, rEspP2, and rApd. **a** Expression of rEspP1, rEspP2, and rApd in *E. coli* BL21 (DE3). MW, molecular weight. Lane 1, Transformants including vectors; Lane 2, Non-induced transformants including plasmids pET-espP1, pET-espP2, and pET-apd; Lane 3, Induced transformants including plasmids pET-espP1, pET-espP2, and pET-apd; Lane 4, Supernatants from the sonication of the induced transformants; Lane 5, Pellets from the sonication of the induced transformants. Asterisk indicates the target protein bands. **b** Purification of the rEspP1, rEspP2, and rApd. Lane 1, Non-binding effluent fraction when loading; Lane 2–8, Eluted fraction using elution buffer containing imidazole at 5, 20, 50, 100, 150, 200, and 300 mM. Asterisks indicate the target protein bands. **c** rEspP1, rEspP2, and rApd were used to coat ELISA plates at 1 μg/ml to detect 12 positive and 12 negative porcine sera of *H. parasuis*. The line represents the average OD_630_ value of the positive sera, and the dotted line represents that of the negative sera. For rEspP1 and rEspP2, the OD_630_ values of negative sera were comparable with those of positive sera (*P* > 0.05), whereas rApd clearly discriminated the positive from negative samples according to the OD_630_ value (*P* < 0.001)

The genes for the passenger domains of three autotransporters of *H. parasuis* were cloned and expressed in *E. coli* BL21 (DE3). The rEspP1 and rEspP2 were approximately 68 and 60 kDa as determined by SDS-PAGE, whereas rApd was approximately 85 kDa (Fig. 1a). The purified proteins were prepared using elution buffer containing 150 mM imidazole for rEspP1 and rEspP2, and 100 mM imidazole for rApd (Fig. 1b). rEspP1, rEspP2, and rApd were used to coat ELISA plates to detect 12 *H. parasuis-*positive and -negative serum samples from vaccinated and non-vaccinated swine herds (Table 1). For rEspP1 and rEspP2, the OD_630_ values of negative sera were comparable with those of positive sera (*P* > 0.05), whereas rApd clearly discriminated the positive from negative samples according the OD_630_ value (*P* < 0.001) (Fig. 1c). Therefore, rApd was selected as the diagnostic antigen for antibody detection ELISA of *H. parasuis*.

**Table 1:** Serum samples from vaccinated and experimentally infected pigs

	Vaccination or infection (No.)	Pigs with signs and lesions following challenge (No.)	Pigs without signs or lesions following challenge (No.)
Pig test 1	PBS control (15)	12 ^*a*^	8
	Commercial killed vaccine (15)	3	12 ^*b*^
	*H. parasuis* 011D (4)	0	4 ^*c*^
Pig test 2	PBS control (4)	3	1
	Commercial killed vaccine (4)	1	3^*d*^
	*H. parasuis* 016B (4)	0	4 ^*e*^
	*H. parasuis* 014H (4)	0	4 ^*f*^

^*a*^ The 12 serum samples were used as negative sera of *H. parasuis* for screening of antigens

^*b*^ The 12 serum samples were used as positive sera of *H. parasuis* for screening of antigens

^*b, d*^ The 15 serum samples from two groups were used as positive sera of *H. parasuis* for evaluation of positive percent agreement of the rApd ELISA

^*c, e, f*^ These 12 serum samples from three groups were used as positive sera of *H. parasuis* for evaluation of positive percent agreement of the rApd ELISA

### Evaluation of rApd as the diagnostic antigen

**Fig. 2:**
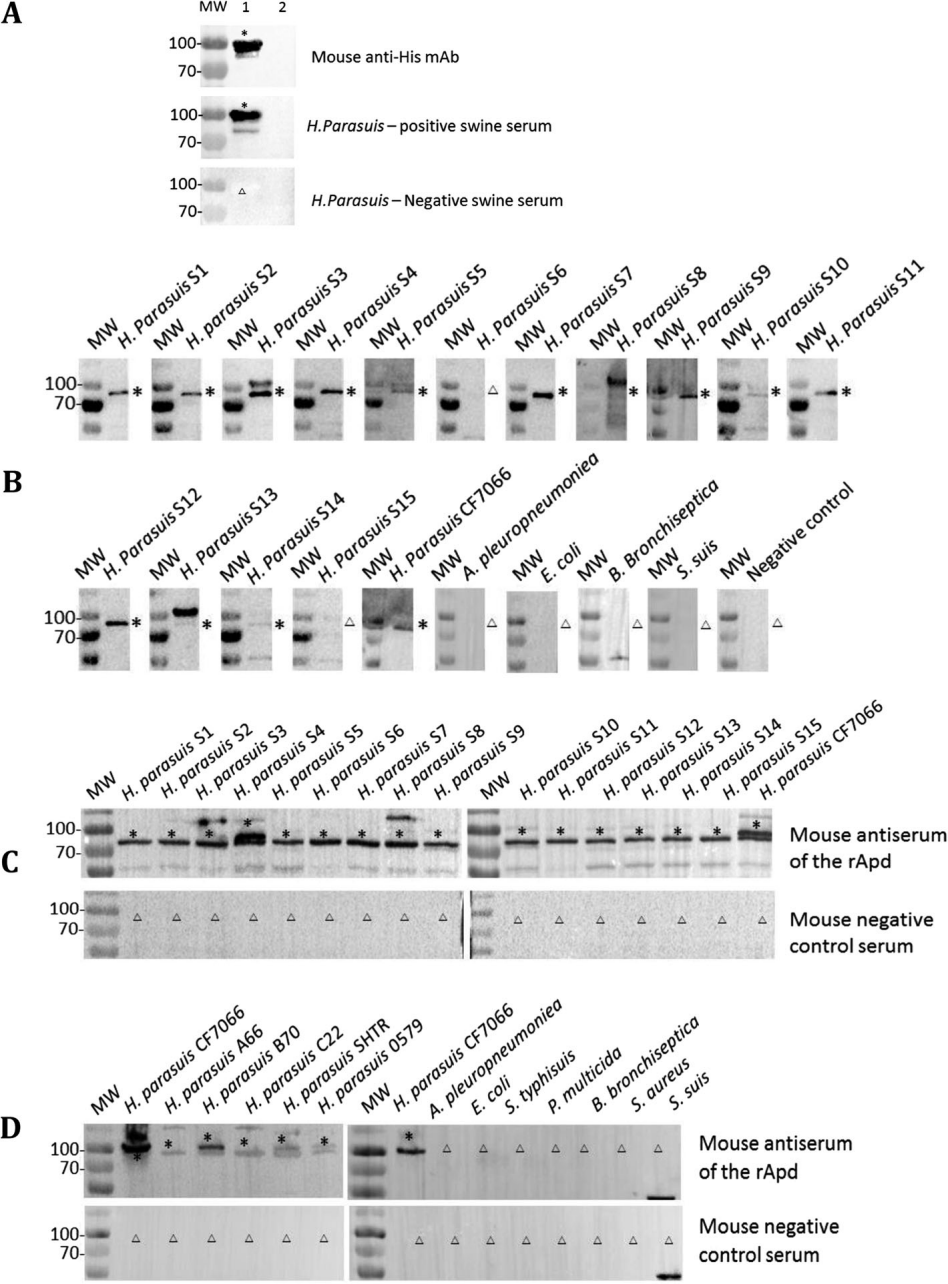
Western blotting analysis of the specificity of purified rApd. **a** Purified rApd was used as antigen and primary antibodies were the anti-histidine monoclonal antibody and *H. parasuis*-positive pig serum. Lane 1, Purified rApd; Lane 2, Purified fraction from a transformant including vector pET-25b. **b** Purified rApd was used as antigen, and primary antibodies were antisera of 15 serovar *H. parasuis* reference strains, isolate CF7066, and four other porcine pathogenic organisms. **c** The rApd antiserum was used as primary antibody, and antigens were bacterial proteins of 15-serotype *H. parasuis* reference strains and isolate CF7066. **d** The rApd antiserum was used as primary antibody, and antigens were five non-typeable *H. parasuis* isolates and seven other porcine pathogenic organisms. MW, molecular weight. *H. parasuis* S1-S15, *H. parasuis* reference strains of serotype 1–15. Asterisks and triangles indicate negative and positive results, respectively

Immunoblotting assays indicated that the purified rApd was recognized by a murine anti-His antibody and a positive porcine serum of *H. parasuis*, but not by a negative porcine serum (Table 1; Fig. 2a). The mouse antisera of *H. parasuis* 15 serotype reference strains and isolate CF7066 reacted with the rApd via western blotting except for serotype 6 and 15 strains. However, the mouse antisera against *A. pleuropneumoniae, E. coli, B. bronchiseptica and S. suis did not react with rApd* (Fig. 2b). Furthermore, the mouse antiserum of rApd reacted with the bacterial proteins of 15 serotype reference strains and isolate CF7066, five non-typeable strains, but not with the seven pathogenic bacteria including *A. pleuropneumoniae*, *E. coli, S. typhi-suis*, *P. multocida*, *B. bronchiseptica*, *S. aureus*, and *S. suis* (Fig. 2 c, d). The results indicated that rApd was universal across 15 serotype and non-typeable strains as well as specific for *H. parasuis*.

### Positive and negative percent agreement of the rApd ELISA

The intact whole cells and supernatant of the sonicated cells of *H. parasuis* were used as separate antigens of ELISA to examine the swine positive and negative sera. The former yielded maximum discrimination between positive and negative serum samples with bacterial suspensions at 0.5 × 10^8^ cfu/mL, and the largest the value of positive/negative ratio was 2.470 (Additional file 1: Figure S1). Therefore, we defined an OD_630_ value of less than 0.47 as indicating negativity for the whole cell ELISA. A total of 330 clinical sera from five farms were measured and 40 tested negative. Among these, 4 samples derived from 25 sucking pigs, 31 from 180 weanling pigs, and 5 from 74 fattening pigs. No negative samples were detected among 50 samples of sows. These 40 sera were accordingly used as *H. parasuis* negative samples (Table 2).

**Table 2:** Measurement of clinical serum samples with rApd and whole cell ELISA

Serum origin	No. of samples	No. of positive samples (%)	
		rApd ELISA	Whole cell ELISA
Suckling pigs	25	2 (12.0%) ^*a*^	21 (84.0%) ^*b*^
Weanling pigs	181	5 (2.8%)	150 (82.9%)
Fattening pigs	74	24 (32.4%)	69 (93.2%)
Sows	50	27 (54.0%)	50 (100.0%)
Total	330	59 (17.8%)	290 (87.9%)

^*a*^ The rApd ELISA showed the numbers of positive samples and positive rate in the same age pigs

^*b*^ Whole cell ELISA showed the numbers of positive samples and positive rate in the same age pigs

The 15 vaccinated pigs and the 12 pigs infected with low virulence strains obtained protective immunity, because they did not exhibit any clinical signs of Glässer’s disease following the challenge of *H. parasuis*. Therefore, 27 serum samples from these pigs were considered to be antibody positive for *H. parasuis* (Table 1).

The 40 negative and 27 positive samples were measured using the optimized rApd ELISA (Additional file 1: Table S3). A ROC curve was built on the basis of the absorbance values of the 67 sera at 630 nm. The OD_630_ value for the negative group was 0.220 ± 0.241, and that for positive serum samples was 1.110 ± 0.345. An AUC value of 0.961 indicated that the assay produced accurate results. The ROC-optimized cutoff value was 0.569 [16]. According to the cutoff value, the rApd ELISA exhibited positive percent agreement values of 96.4% and negative percent agreement values of 94.9%. And area under the ROC curve (AUC) value of 0.961 indicated the assay produced accurated results (Additional file 1: Figure S2).

To assess the reproducibility of the rApd ELISA, 15 serum samples including five negative, five weakly positive, and five strongly positive were measured six times to determine intra-assay repeatability. The CV value of intra-assay repeatability was between 3.8 and 6.6%. These 15 sera were also tested using three patches of rApd antigens, indicating that inter-assay repeatability was between 0.3 and 10.3% (Additional file 1: Table S3). These results indicate the rApd ELISA had good reproducibility.

### Measurement of serum samples from experimentally vaccinated or infected pigs

**Fig. 3:**
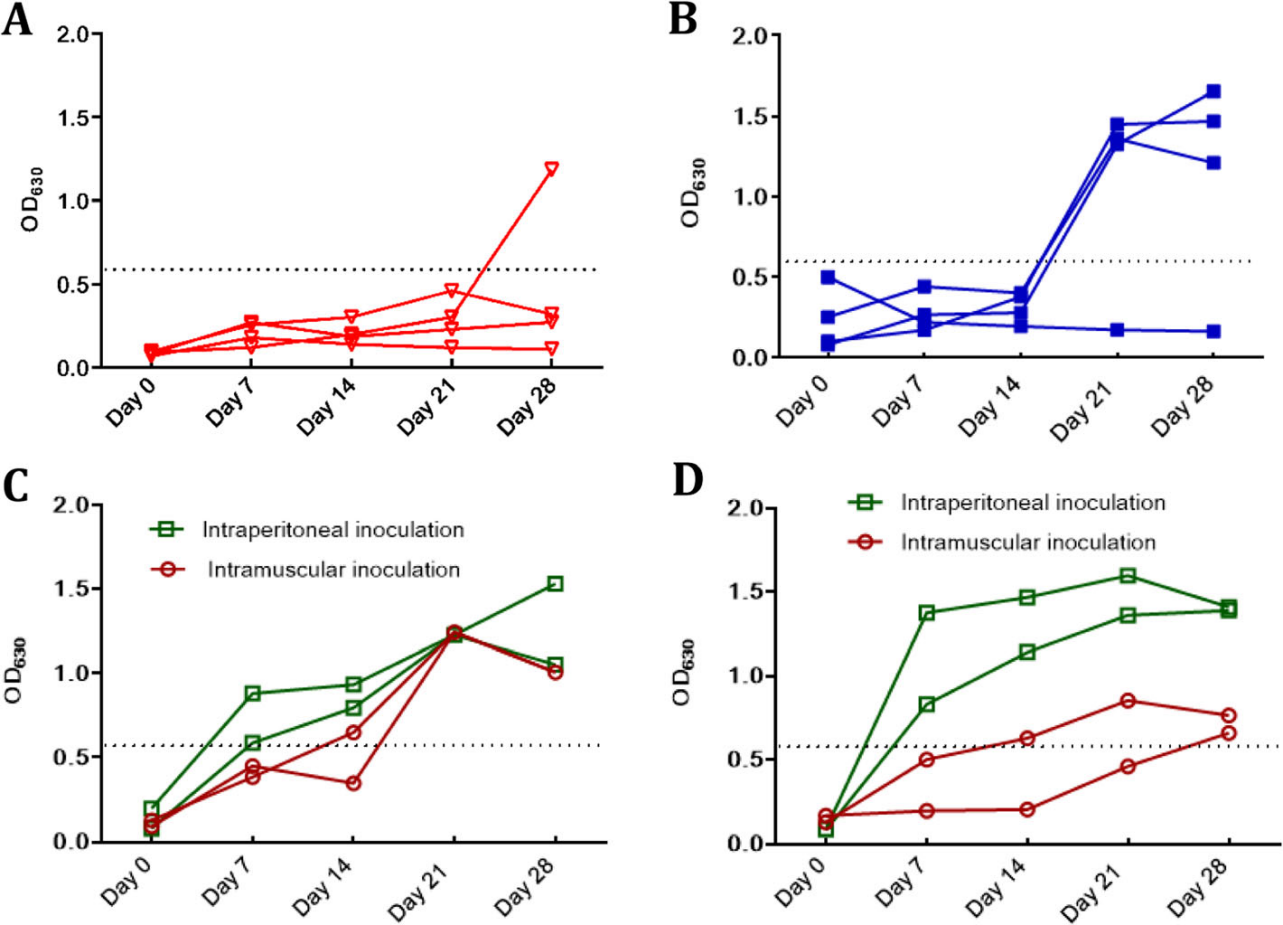
Detection of experimentally vaccinated and infected pigs using the rApd-ELISA. **a** PBS group. **b** Group immunized with the commercial killed vaccine. **c** Group infected with low virulence strains 016B either intraperitoneally or muscularly. **d** Group infected with low virulence strain 014H either intraperitoneally or muscularly. The cutoff value of 0.569 is marked by dotted lines. Green squares and red circles indicate intraperitoneal and intramuscular infection, respectively

The rApd ELISA was used to detect the serologic conversion and duration from experimentally vaccinated or infected pigs. The four pigs in the PBS group remained negative within 4 weeks; only one pig became positive at week four (Fig. 3a). The vaccinated group did not produce an antibody until 1 week after the second vaccination. One pig in the vaccinated group remained seronegative because that pig had been demonstrated to support an unknown bacterium colonizing its nasal cavity at the beginning of the trial (Fig. 3b). The two groups infected with isolates 016B and 014H via intraperitoneal injection, began to raise antibodies at day seven after the first infection. However, the pigs infected muscularly showed a similar antibody production profile as that of the vaccinated pigs (Fig. 3c and d).

### Measurement of serum samples from clinically healthy pigs

A total of 330 sera samples from clinically healthy herds without vaccination were tested using rApd to obtain a 12.0, 2.8, 32.4, and 54.0% positive rate in the suckling pigs, weanling pigs, fattening pigs and sows, respectively; the total positive rate was 17.9% (59/330). However, the whole cell ELISA acquired the positivity ratios of 84.0, 82.9, 93.2 and 100% in the suckling pigs, weanling pigs, fattening pigs, and sows (total positive rate of 87.9% (290/330)) (Table 2).

## Discussion

Among *H. parasuis* autotransporters, one monomeric autotransporter, Apd, was identified as a suitable antigen for an antibody detection ELISA of *H. parasuis*. The purified rApd was confirmed to be specific to *H. parasuis* using immunoblotting assays (Fig. 2). The antibodies induced by *H. parasuis* commercial killed vaccine and infection were both detectable via the rApd ELISA (Fig. 3), which is consistent with the findings that autotransporters of *H. parasuis* were able to be expressed in in vitro culture as well as in in vivo infection of pigs [15]. In addition, Apd was present in 15 serotype reference strains and five non-typeable strains tested; thus, the rApd ELISA exhibited the potential to be used for antibody detection to determine vaccine immunity and serological diagnosis of Glässer’s disease regardless of the serotype of strains. Additionally, rApd was identified as a valid antigen using the sera from pigs that had survived challenge; therefore, positivity for the rApd ELISA might be associated with protective immunity. However, it does not discriminate immunization sera from infection sera because Apd could be expressed both in vivo and in vitro. Therefore, other antigens need to be screened for the development of a serological method to discriminate infection from immunity in Glässer’s disease.

Among three autotransporter passenger domains, EspP1 and EspP2 did not discriminate the negative and positive porcine sera (Fig. 1c). Protein BLAST in NCBI suggested that EspP1 and EspP2 shared homologous amino acid sequence with the autotransporters of *Campylobacter* species, with highest similarity of 51% identity under 91% overall coverage. Among these, *Campylobacter lanienae* and *Campylobacter hyointestinalis* could be isolated from healthy pigs [17, 18]. Apd was only found to be homologous with a metallo-endopeptidase of *S. suis*, with 27% identity under 54% coverage for the most similar sequence. However, no reaction was observed in western blotting between rApd and *S. suis* antiserum, or between *S. suis* bacterial proteins and the rApd antiserum (Fig. 2). We hypothesized that the low identity and coverage between the two bacterial proteins was not sufficient to form a highly similar structure to lead to cross reactivity.

In the present study, only 27 positive sera were used to evaluate the positive percent agreement as we were unable to achieve other methods to identify serum background. The positive sera were all derived from pigs that obtained protective immunity after vaccination and infection. The 40 negative sera were obtained by screening a panel of 330 serum samples of clinically healthy pigs using the *H. parasuis* whole-cell ELISA. Whole cells usually result in high sensitivity and low specificity. If samples tested negative by the whole-cell ELISA, they were considered to be true negative [19]. Additionally, negative samples were screened from clinically healthy pigs rather than colostrum-deprived piglets because the former represented the diversity and complexity of serum samples of different herds, unlike the latter that tended to yield very low OD_630_ values.

Antibody detection assays over 4 weeks indicated that antibodies appeared 1 week after the first infection, albeit at 1 week after the second vaccination (Fig. 3)*.* This indicated that the infection elicited detectable antibodies faster than vaccine immunization. The complement fixation test with heat-treated whole cells as antigens also suggested that the seroconversion for a bivalent killed vaccine arises at day 19 after the second immunity [9]. Pigs infected once intramuscularly were almost negative within 2 weeks, whereas the pigs infected once intraperitoneally rapidly raised antibodies in 1 week and remained at a high OD_630_ value (Fig. 3c and d). This difference may have occurred because intraperitoneal infection likely led to bacterial cell replication, whereas muscular infection only provided bacterial antigens as immunogens. Additionally, the time and level of antibody production also differed for 016B and 014H even though they both were infected intraperitoneally (Fig. 3c, d), which might be related to the invasion capability of strains and intensity of the elicited inflammation and immunity response.

Apd was screened and identified as a diagnostic antigen in ELISA for *H. parasuis* antibody detection, and the rApd ELISA was confirmed to be able to detect the production and duration of serum antibodies of experimentally infected and vaccinated pigs. However, it remains to be evaluated with serum samples of animals suffering from Glässer’s disease and those immunized with commercially available vaccines containing killed pathogens in field conditions.

## Conclusions

The present study identified *H. parasuis-specific* Apd as a novel diagnostic antigen. An indirect ELISA based on the rApd was developed and demonstrated to be able to detect antibodies elicited by both infection and vaccination of *H. parasuis*. Detectable antibodies appeared 1 week after infection albeit 2 weeks after vaccination with the commercial killed vaccine. This ELISA thus exhibits the potential to be applied toward Glässer’s disease diagnosis, vaccine efficacy evaluation, and serological surveillance of H. parasuis infection.

## Methods

### Materials

*H. parasuis* strains were grown on tryptic soy broth or tryptic soy agar (TSB or TSA, Difco, Detroit, MI, USA) supplemented with 10 μg/ml NAD and 5% bovine serum. A. pleuropneumoniae, Escherichia coli, Salmonellatyphisuis, Pasteurella multocida, Bordetella bronchiseptica, Staphylococcus aureus, and S. suis were grown on TSB or TSA supplemented with 5% bovine serum. When required, media were supplemented with kanamycin (50 μg/mL) or ampicillin (100 μg/mL) (Additional file 1: Table S1).

The passenger domains of three autotransporter genes were amplified from *H. parasuis* isolate CF7066. The primer pairs for two paralogous extracellular serine proteases (Esp) and Apd were designed (Additional file 1: Table S2). The PCR products of *esp*P1 and *esp*P2 (MK617355 and MK617356) were ligated into the expression vectors pET-28a and pET-25b (Novagen, Madison, WI, USA) to construct the plasmids pET-espP1 and pET-espP2. The PCR fragment of gene *apd* (MK617354) was cloned into vector pET-25b to construct plasmid pET-apd.

### Preparation of recombinant proteins EspP1, EspP2, and Apd

The three plasmids pET-espP1, pET-espP2 and pET-apd were used to transform *E. coli* BL21 (DE3)*.* EspP1, EspP2, and Apd were expressed as His fusion proteins in *E. coli* BL21 (DE3) and purified using Ni Sepharose 6Fast Flow (GE Healthcare Biosciences, Pittsburgh, PA, USA). The three purified protein concentrations were measured using a BCA kit (Bioshap, Hefei City, China).

### Serum from mice and pigs

Pig and mouse experiments were conducted in accordance with the recommendations in the Guide for the Care and Use of Laboratory Animals Centre of Huazhong Agricultural University. Experimental procedures for pigs and mice were approved by the Scientific Ethic Committee of Huazhong Agricultural University (No. HZAUSW-2017-009 and No. HZAUMO-2018-028).

Fifty-five healthy ternary hybrid pigs at five-weeks-of-age were purchased from the conventional farms that had no recorded instance of Glässer’s disease. *H. parasuis*-whole cell ELISA was used to screen serum-negative pigs. Following transferring to Laboratory Animals Centre of Huazhong Agricultural University, they were raised in isolated and ventilated animal rooms. Forty-two BALB/c female mice at five-weeks-of-age were purchased from and housed in Laboratory Animals Centre of Huazhong Agricultural University, and were raised under specific pathogen free conditions.

In pig test 1, a total of 39 healthy ternary hybrid pigs were used. Twenty pigs were injected with phosphate buffered saline (PBS) as a negative control, 15 pigs were immunized with a commercial killed vaccine of *H. parasuis,* and the remaining 4 pigs were infected with low virulence isolate 011D at an amount of 1 × 10^9^ colony-forming units (CFU). Three groups were housed separately in isolated and ventilated animal rooms. Vaccination or infection was implemented twice with an interval of 2 weeks. All pigs were bled 2 weeks after the second immunization or infection and then challenged with *H. parasuis* virulent strain SH0165 at 2 × 10^10^ CFU under anesthesia. The pigs were euthanized with an intravenous overdose of sodium pentobarbital (100 mg/kg) for necropsy at 7 days post infection (dpi) if they did not die from the infection or within 7 dpi when they presented characteristic signs of Glässer’s disease (Table 1).

In pig test 2, a panel of 16 healthy pigs at five-weeks-of-age was used. Animals were randomly divided into four groups and housed separately in isolated and ventilated animal rooms. PBS, the commercial killed vaccine was injected intramuscularly, and low virulence H. parasuis strains 016B and 014H were infected at an amount of 1 × 10^9^.

CFU, with two pigs infected intramuscularly and intraperitoneally in each group. All pigs were challenged with *H. parasuis* virulent strain SH0165 at 2 × 10^10^ CFU and euthanized with an intravenous overdose of sodium pentobarbital (100 mg/kg) at 7 days after challenge for necropsy (Table 1).

A total of 42 BALB/c female mice at five-weeks-of-age were used for mouse antiserum preparation. Two mice were initially immunized with purified rApd (100 μg) emulsified 1:1 in complete Freund’s adjuvant by subcutaneous injection. After 2 weeks, a second immunization was administered with 100 μg purified rApd emulsified 1:1 in incomplete Freund’s adjuvant. A booster was administered 2 weeks later. Mice were euthanized by intraperitoneal injection of overdose of sodium pentobarbital (100 mg/kg). Serum was collected on the third day after the booster immunization. Thirty-eight mice were used for preparation of antiserum of 15-serotype reference strains and other four porcine bacterial pathogens using the same procedure. Two mice were injected with (PBS) as a negative control.

Additionally, a total of 330 sera of clinically healthy pigs were collected from five farms, including sucking pigs, weanling pigs, fattening pigs, and sows (Table 2).

### Indirect ELISA

Indirect ELISA was performed according to the basic protocol. After coating in carbonate buffer (pH 9.6) [Na_2_CO_3_ 15 mM, NaHCO_3_ 35 mM] overnight at 4 °C, ELISA plates were blocked with PBST (PBS including 0.05% Tween 20) containing 5% skim milk at 37 °C for 2 h. After washing three times with PBST, sera were diluted 1:100 with PBST containing 0.5% bovine serum albumin (BSA), added to wells and incubated for 30 min at 37 °C. After washing three times, HRP-labeled goat anti-pig IgG (Jackson Immuno Research Laboratories, West Grove, PA, USA) was diluted 1:10,000 in PBST containing 0.5% BSA and added to plates, followed by incubation at 25 °C for 30 min. After washing three times, tetramethylbenzidine (TMB) peroxidase substrate and peroxidase H_2_O_2_ were added to each well to incubate at 25 °C for 15 min away from light. The reaction was quenched by adding hydrofluoric acid, and the optical density at 630 nm (OD_630_) was measured using an ELISA reader (Tecan, San Jose, CA, USA).

After the rApd was selected as an optimal antigen, checkerboard titration was performed to determine the optimal dilution of antigen, serum, and secondary antibody. The purified Apd was diluted to 0.5 μg/ml in carbonate buffer (pH 9.6) to coat ELISA plates overnight at 4 °C, sera were diluted at ratio of 1:200 and secondary antibody were diluted 1:15,000.

*H. parasuis-*whole cell ELISA was developed as follows. The intact cells and the supernatant of sonicated *H. parasuis* CF7066 (serotype 5) cells were prepared and used to coat ELISA plates. The bacterial culture and supernatant of the sonicated cells was diluted in 2-fold serial dilutions. *H. parasuis* positive and negative sera were detected in order to determine the antigen forms and concentration for *H. parasuis* whole cell ELISA.

### Immunoblotting

The rApd was examined by western blot using anti-His monoclonal antibodies (ABclonal), porcine positive serum against *H. parasuis*, and murine sera against 15 serotype reference strains of *H. parasuis* and other five porcine pathogenic bacteria. The purified rApd was subjected to sodium dodecyl sulfate-polyacrylamide gel electrophoresis (SDS-PAGE) and transferred to nitrocellulose membranes. The membranes were blocked with TBST (20 mM Tris-HCl, 150 mM NaCl, 0.05% Tween 20, pH 7.4) containing 5% skim milk. After washing three times with TBST, the membranes were cut apart and each strip was incubated with each bacterial antiserum at a dilution of 1:5000 (anti-His) or 1:100 (swine or murine sera) in TBST containing 5% skim milk. After washing three times with TBST, the membranes were incubated with horseradish peroxidase (HRP)-labeled goat against-mouse IgG (ABclonal) in a 1:5000 dilution in TBST containing 5% skim milk. After washing three times, the bound antibodies were developed for color using Clarity Western ECL Substrate (Bio-Rad, Hercules, CA, USA). Recognition of the native Apd in *H. Parasuis* strains and potentially similar proteins in seven pathogenic bacteria using mouse rApd antiserum were conducted as the same procedure.

### Data analysis

The optimal cutoff value for the rApd ELISA with 95% confidence interval (CI) was established by receiver operating characteristic (ROC) curve analysis using the EpiTools epidemiological calculator (http://epitools.ausvet.com.au) [16]. The area under the curve (AUC) values was considered as follows: noninformative, AUC = 0.5; low accurate, 0.5 < AUC ≤ 0.7; moderately accurate, 0.7 < AUC ≤ 0.9; highly accurate, 0.9 < AUC < 1; perfect, AUC = 1 [20].

## Supplementary information

**Additional file 1.** Supplementary materials for Development and application of an antibody detection ELISA for Haemophilus parasuis based on a monomeric autotransporter passenger domain.

**Additional file 2.** The ARRIVE Guidelines Checklist for Animal Research: Reporting In Vivo Experiments.

## Data Availability

The sequencing data generated during this study are available in the GenBank repository, http://www.ncbi.nlm.nih.gov. The accession numbers are MK617354, MK617355 and MK617356.

## *Abbreviations*

Apd: Autotransporter passenger domain
Esp: Extracellular serine protease
CFU: Colony-forming unit
dpi: Days post infection
PBS: Phosphate buffered saline
AUC: Area under the ROC curve
ROC: Receiver operating characteristic
