# Supplementary material for: Development and application of an antibody detection ELISA for Haemophilus parasuis based on a monomeric autotransporter passenger domain
Source: BMC Vet Res. 2019 Dec 3;15:436. doi: 10.1186/s12917-019-2128-x (PMC6891974; doi:10.1186/s12917-019-2128-x)
Supplement: Supplementary file 1 — Additional file 1. Supplementary materials for Development and application of an antibody detection ELISA for Haemophilus parasuis based on a monomeric autotransporter passenger domain. [file 12917_2019_2128_MOESM1_ESM.docx]

**Table S1** Strains and plasmids used in this study.

| **Strain** | **Relevant characteristic or description** | **Source** |
| --- | --- | --- |
| *H. parasuis* NR4 | Serotype 1 reference strain | Japan |
| *H. parasuis* SW140 | Serotype 2 reference strain | Japan |
| *H. parasuis* SW114 | Serotype 3 reference strain | Japan |
| *H. parasuis* SW124 | Serotype 4 reference strain | Japan |
| *H. parasuis* Nagasaki | Serotype 5 reference strain | Japan |
| *H. parasuis* 131 | Serotype 6 reference strain | Switzerland |
| *H. parasuis* 174 | Serotype 7 reference strain | Switzerland |
| *H. parasuis* C5 | Serotype 8 reference strain | Sweden |
| *H. parasuis* D74 | Serotype 9 reference strain | Sweden |
| *H. parasuis* H367 | Serotype 10 reference strain | Germany |
| *H. parasuis* H465 | Serotype 11 reference strain | Germany |
| *H. parasuis* H425 | Serotype 12 reference strain | Germany |
| *H. parasuis* IA-84-17975 | Serotype 13 reference strain | USA |
| *H. parasuis* IA-84-22113 | Serotype 14 reference strain | USA |
| *H. parasuis* SD-84-15995 | Serotype 15 reference strain | USA |
| *H. parasuis* SH0165 | Serotype 5 isolate, for challenge | This work |
| *H. parasuis* CF7066 | Serotype 5 isolate, for gene cloning | This work |
| *H. parasuis* A66 | Non-typeable isolate | This work |
| *H. parasuis* B70 | Non-typeable isolate | This work |
| *H. parasuis* C22 | Non-typeable isolate | This work |
| *H. parasuis* SHTR | Non-typeable isolate | This work |
| *H. parasuis* 0579 | Non-typeable isolate | This work |
| *H. parasuis* 011D | Serotype 5 isolate for pig test 1 | This work |
| *H. parasuis* 016B | Serotype 5 isolate for pig test 2 | This work |
| *H. parasuis* 014H | Serotype 4 isolate for pig test 2 | This work |
| *Actinobacillus pleuropneumoniae* | Isolate in our laboratory | This work |
| *Escherichia coli* | Isolate in our laboratory | This work |
| *Pasteurella multocida*, | Isolate in our laboratory | This work |
| *Bordetella bronchiseptica* | Isolate in our laboratory | This work |
| *Streptococcus suis* | Isolate in our laboratory | This work |
| pET-25b | *E. coli* expression vector, His Tag, Amp^R^ | Novagen |
| pET-28a | *E. coli* expression vector, His Tag, Kan^R^ | Novagen |
| pET-apd | Expression plasmid of autotransporter passenger domain (Apd) constructed from pET-25b | This work |
| pET-espP1 | Expression plasmid of putative extracellular serine protease 1 constructed from pET-28a | This work |
| pET-espP2 | Expression plasmid of putative extracellular serine protease 2 constructed from pET-25b | This work |

**Table S2** Primers used in this study.

| **Primers** | **Primer sequence (5′→3′)** |
| --- | --- |
| apd-F | CG*gaattc* (EcoRI) GCAAACGTATACTGTTACAGGTAGC |
| apd-R | CC*ctcgag* (XhoI) GGTGATTGTGATTTTATTGTGGT |
| espP1-F | CC*ctcgag* (XhoI) TTTCGTGATATGGATATTTCCAT |
| espP1-R | CG*gaattc* (EcoRI**)** GATGTCTACTGGGCAAGTG |
| espP2-F | CG*gaattc* (EcoRI)CAGACTTATTGGGCAAGTG |
| espP2-R | CC*ctcgag* (XhoI) TTTCGTGATATGGATATTTCCA |

**Table S3** Intra-assay and inter-assay reproducibility.

| Serum samples (No.) | | Intra-assay variability | | Inter-assay variability | |
| --- | --- | --- | --- | --- | --- |
|  |  | OD_630_  Mean ± SD (n=6) | CV% | OD_630_  Mean ± SD (n=3) | CV% |
| Negative | 1(C59) | 0.308 ± 0.020 | 6.6 | 0.339 ± 0.027 | 8.0 |
|  | 2(42) | 0.256 ± 0.013 | 5.0 | 0.171 ± 0.018 | 10.3 |
|  | 3(40) | 0.241 ± 0.018 | 7.7 | 0.179 ± 0.016 | 9.1 |
|  | 4(38) | 0.479 ± 0.026 | 5.4 | 0.452 ± 0.031 | 6.8 |
|  | 5(37) | 0.265 ± 0.014 | 5.4 | 0.386 ± 0.007 | 1.8 |
| Weakly positive | 6(H) | 0.907 ± 0.039 | 4.2 | 1.118 ± 0.036 | 3.3 |
|  | 7(91) | 1.030 ± 0.045 | 4.4 | 1.287 ± 0.004 | 0.3 |
|  | 8(90) | 0.970 ± 0.042 | 4.3 | 1.038 ± 0.031 | 3.0 |
|  | 9(36) | 1.068 ± 0.048 | 4.5 | 1.018 ± 0.005 | 0.5 |
|  | 10(31) | 0.877 ± 0.036 | 4.1 | 0.855 ± 0.047 | 5.5 |
| Strongly positive | 11(G) | 1.890 ± 0.121 | 6.4 | 2.065 ± 0.112 | 5.4 |
|  | 12(28) | 1.660 ± 0.115 | 7.0 | 1.611 ± 0.080 | 5.0 |
|  | 13(17) | 1.951 ± 0.094 | 4.8 | 1.892 ± 0.124 | 6.5 |
|  | 14(3) | 1.948 ± 0.073 | 3.8 | 1.985 ± 0.019 | 1.0 |
|  | 15(1) | 1.816 ± 0.074 | 4.1 | 1.966 ± 0.117 | 6.0 |

**
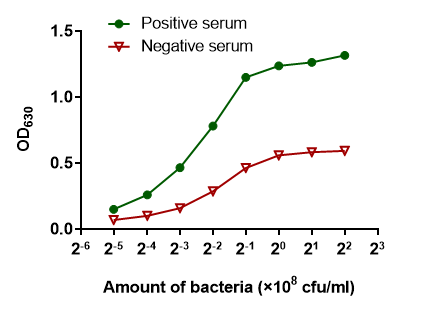
**

**A**

**B**


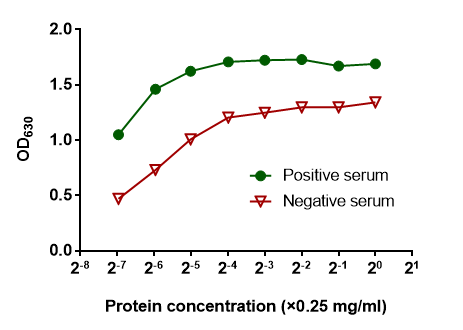


**B**

**Figure S1** Identification of coating antigens for *H. parasuis* whole cell ELISA. (A) The

bacterial suspension was diluted in 2-fold serial dilutions, from 2^2^ × 10^8^ to 2^−5^ × 10^8^

CFU/mL. (B) The supernatant of the sonicated *H. parasuis* cells was diluted in 2-fold serial dilutions, from 1 to 2^−7^ mg/mL. The positive and negative sera were from experimentally vaccinated and non-vaccinated pigs.


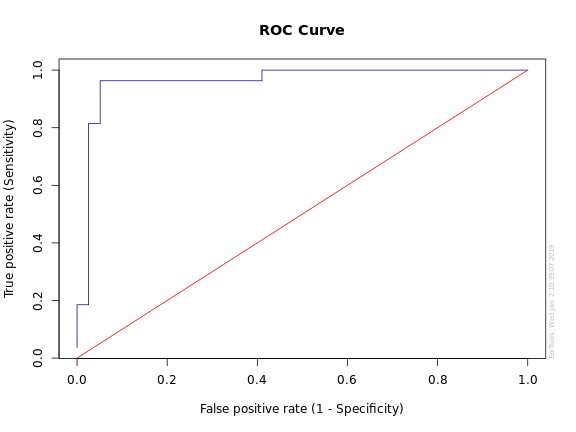


**Figure S2** The receiver operating characteristic (ROC) curve was constructed using 40 negative serum samples from healthy pigs at different ages in four farms and 27 positive serum samples from the pigs that were vaccinated and infected and had obtained protective immunity. The value for the area under the ROC curve (accuracy) was 0.961.
