# Supplementary material for: Development and application of an antibody detection ELISA for Haemophilus parasuis based on a monomeric autotransporter passenger domain
Source: BMC Vet Res. 2019 Dec 3;15:436. doi: 10.1186/s12917-019-2128-x (PMC6891974; doi:10.1186/s12917-019-2128-x)
Supplement: Supplementary file 2 — Additional file 2. The ARRIVE Guidelines Checklist for Animal Research: Reporting In Vivo Experiments. [file 12917_2019_2128_MOESM2_ESM.pdf]

Dear GenBank Submitter:

Thank you for your submission.

Based on the data submitted to us, the scheduled release date for your submission is:

Sep 29, 2019

If this date is not correct, please let us know as soon as possible, otherwise this submission will be released on the date indicated above. The data will become available from our different servers within a few days of release. The data are simultaneously made available to ENA in Europe and the DNA Data Bank of Japan.

Changes may have been made to your original submission in order to conform to database annotation conventions including:

- Strings of N's, low quality sequence, vector or linker trimmed from ends
- Feature spans corrected and descriptions modified for all feature types including coding regions
- Nomenclature edited to 'official' gene names, product labels, etc.
- Exon spans adjusted to conform to the splice donor/acceptor consensus sequences, GT and AG, respectively
- Any mRNA or ribosomal RNA sequences submitted on the minus-strand have been reverse-complemented
- Taxonomic and source data edited, including unpublished organism names changed to temporary names. Please notify us when the organism names are published and we will update them accordingly.

If your submission needs revision, do not submit a new sequence. Instead, please follow the directions to update a sequence record at: https://www.ncbi.nlm.nih.gov/Genbank/update.html Since the flatfile record is a display format only and is not an editable format of the data, do not make changes directly to a flatfile. Send properly formatted updates to: gb-admin@ncbi.nlm.nih.gov

An accession number has been assigned to each nucleotide sequence and was previously provided to you. Note that during the processing, we have

assigned protein identifiers to any proteins within the submission. This
is fielded as /protein_id.

We strongly recommend that these numbers appear in any publication which reports or discusses these data, so that readers may easily retrieve your data from our databases.

Please reply using the current Subject line.

Sincerely,

Linda Yankie, PhD
GenBank Direct Submission Staff
gb-admin@ncbi.nlm.nih.gov

GenBank flat file:

```
LOCUS       MK617354                1494 bp    DNA     linear
BCT 23-SEP-2019
DEFINITION  Glaesserella parasuis strain CF7066 Apd gene, partial cds.
ACCESSION   MK617354
VERSION     MK617354
KEYWORDS    .
SOURCE      Glaesserella parasuis
  ORGANISM  Glaesserella parasuis
            Bacteria; Proteobacteria; Gammaproteobacteria; Pasteurellales;
            Pasteurellaceae; Glaesserella.
REFERENCE   1  (bases 1 to 1494)
  AUTHORS   Xu,X.
  TITLE     Direct Submission
  JOURNAL   Submitted (11-MAR-2019) Veterinary Infectious Diseases, College
of
            Veterinary Medicine, Huazhong Agricultural University, Shizishan
            Street 1#, Wuhan, Hubei Province 430070, China
COMMENT     ##Assembly-Data-START##
            Sequencing Technology :: Sanger dideoxy sequencing
            ##Assembly-Data-END##
FEATURES             Location/Qualifiers
     source          1..1494
                     /organism="Glaesserella parasuis"
                     /mol_type="genomic DNA"
                     /strain="CF7066"
                     /serotype="5"
```

/isolation_source="Glasser's disease outbreak farm"
/db_xref="taxon:738"
/country="China"
/PCR_primers="fwd_name: apd-f, fwd_seq: caaacgtatactgttacaggtagc, rev_name: apd-r, rev_seq: ggtgattgtgattttattgtggt"

CDS <1..>1494
/note="the passenger domain of the autotransporter"
/codon_start=1
/transl_table=11
/product="Apd"
/protein_id="QES71295"

/translation="QTYTVTGSRLDGDTQLLRGSGFFNVDKQVTIDGQGNRLHLEGYN

LSLGQDVTFSNVNLSLKPSRDLNNIFGSGFNNLKAASTYIVTNGNKLILDNVRTNVNG

EPADTRPMILLGNIDPTKNNNGHDALVVTNAHPTETILSSVVVIGNNNSNTDTPVTIS

LGENVSFVNQVIFEREGEEDIYSGAVYLAGMNNDQEHNRAINFSSSSNAITKIYTGEA

TNVSVTLNNINPETAITLQDVKDLTLNNSRITLDKNLSVSETLTLNNQSAITTVASKD

AFGDIAKTSLMLNNIHTNGNNSNITIVRDNALLINGNITGELTINTEDKDNNVSLPNG

STLVGENGSYVVKLKTEVTQPVSENTTTDSSNTDVTPAKPTDSTPATTGEQTGSSDTT

SPTNTAATSNETTPPATTGDTATNSSNTDVPPATPTDSTPATTGEQTGSSDITSPTDT

AQTDDQAEASSSDTATSTPAQPTGDVASTSEQATTSSTSTNHNKITIT"

ORIGIN
1 caaacgtata ctgttacagg tagccgctta gacggagata ctcaattatt gcgtggaagt
61 ggttttttca atgttgataa acaagtaaca attgatggac aaggaaatcg cttacacctt
121 gaaggttata atttgtcctt aggacaagat gttacttttt cgaatgtaaa tttatcctta
181 aaacctagtc gagatttaaa caatattttt ggttctggat ttaataattt aaaagcagca
241 tctacctata ttgtgaccaa tggaaataaa ttgatcttgg ataatgttag aacgaatgtt

301 aatggtgaac ctgcagatac tcgcccaatg attttacttg gaaatattga tccaacaaaa
361 aataataatg gacacgatgc tcttgtagtg acaaatgctc acccaacaga aacaatatta
421 tcttcagtgg ttgttatagg caataacaat tcaaatactg acaccccagt aacgattagt
481 ttaggggaaa atgtcagctt tgttaatcag gtaatatttg agagagaagg agaagaggat
541 atatatagtg gtgcggtgta tctagctgga atgaataatg atcaagaaca taacagagct
601 attaatttct ctagttcatc aaatgctatc actaaaattt atacgggaga agctacaaat
661 gtttctgtta cgttaaataa catcaatcct gaaactgcta tcactttaca agatgttaaa
721 gatttaacat taaataatag ccgtattact ttggataaga atttatctgt ttcagaaacc
781 ttaactctga ataatcaatc cgcaatcact acagtggcga gtaaagatgc ttttggtgac
841 atcgctaaga ctagtttgat gttaaataat atccatacca atggtaataa tagtaatatc
901 actatcgttc gggacaatgc tcttttgatt aatggtaata ttactggtga attgactatt
961 aatacagaag ataaagataa taacgttagt cttcctaatg gcagcacttt agtaggagaa
1021 aatggcagct atgttgtgaa gctaaaaaca gaagtaactc aacctgtatc agaaaataca
1081 actactgaca gcagtaatac tgatgtaacc cctgctaaac ctacggatag cacaccagca
1141 acaacaggcg aacaaactgg ttcgtcagat acaacatcac caacaaatac agctgcaact
1201 agcaatgaaa ctacaccgcc tgctacaacg ggagatacag cgactaacag cagtaatact
1261 gatgtacccc ctgctacacc tacggatagc acaccagcaa caacaggcga acaaactggt
1321 tcgtcagata taacatcgcc aacagataca gctcaaacag acgatcaagc ggaggcttct
1381 agctctgaca ctgcaacatc tacaccagca caaccaacgg gcgatgtagc ttcaacaagt
1441 gaacaagcga caacgtcgag tacatctaca aaccacaata aaatcacaat cacc

//

LOCUS MK617355 1845 bp DNA linear BCT 23-SEP-2019

DEFINITION Glaesserella parasuis strain CF7066 espP1 gene, partial cds.

ACCESSION MK617355

VERSION MK617355

KEYWORDS .

SOURCE Glaesserella parasuis

ORGANISM Glaesserella parasuis
Bacteria; Proteobacteria; Gammaproteobacteria; Pasteurellales;
Pasteurellaceae; Glaesserella.

REFERENCE 1 (bases 1 to 1845)

AUTHORS Xu,X.

TITLE Direct Submission

JOURNAL Submitted (11-MAR-2019) Veterinary Infectious Diseases, College of
Veterinary Medicine, Huazhong Agricultural University, Shizishan
Street 1#, Wuhan, Hubei Province 430070, China

FEATURES Location/Qualifiers

source 1..1845
/organism="Glaesserella parasuis"
/mol_type="genomic DNA"
/strain="CF7066"
/serotype="5"

/isolation_source="Glasser's disease outbreak farm"
/db_xref="taxon:738"
/country="China"
/PCR_primers="fwd_name: espp1-f, fwd_seq: gatgtctactgggcaagtg, rev_name: espp1-r, rev_seq: tttcgtgatatggatatttccat"

CDS <1..>1845
/note="putative extracellular serine protease (autotransporter)"
/codon_start=1
/transl_table=11
/product="espP1"
/protein_id="QES71296"

/translation="DVYWASGVDKQKGWTADFQYPNQCWGAVAGNMLGWWKREL
KTPV

LFNPNTPNDNKEISKWLNKKYPNIQGGLYPFRGMEYFFEDFAPTVKLYETHNT
KTSYE

AQRGPTKISGGPFWTERYDSNAALVTKSLVENFKTGNVVAALTSWTHTVTLW
GIEVDE

KTGKIKKGYISDSVADQAGNLKMVEVTGDYVLDNKGNEIFRFLYSYYVPNGN
KYVTDV

YDIYSITYVSIDETRNNGTYKDTTNREDCKLSLAPGADASYCGISNSTTTTTAT
ENNS

STNTETVAENTNEASTVTTEPAPEETDVDAQSSNTETSETETVSEVPPTKEAVA
ENNE

QIATSNTETTVSEENSSEITSTDSPASNAETVVLETENTPITPTNGNSSEPSVDNT
AQ

SAVEMPVVVTEEDKPTESVVAENNEKPTATSNTETTVSEENSSEITSTDSSASN
AETV

VLETENTPSTPANENSDEPSVDNTAQPTVETPVVATEDAKPTAEAVAETSEQPT
AVPT

PEVAEPAPEPTPSLASTETATVSEVKPTEPETAERLAVKSLKEAIANYQELSFVT
TRV

GDVTRYLIQREGIDIALIDPNSKQILVNNTGLVNYDLAQDQNGNIHITK"
ORIGIN

```
        1 gatgtctact gggcaagtgg agttgataag caaaaaggtt ggactgctga ttttcaatat
       61 cctaaccaat gctggggagc tgttgcagga aatatgcttg gttggtggaa aagagaatta
      121 aaaacccctg ttttatttaa tcccaataca ccaaacgata ataaggagat ttcaaaatgg
      181 ttaaataaga aatatccaaa catacaggga ggattatacc catttcgtgg aatggaatat
      241 ttctttgagg attttgctcc cactgttaag ttatatgaaa ctcataatac gaaaacttct
      301 tatgaggcac agcgtgggcc aacaaaaatc agtggtggtc cattttggac tgagcgttac
      361 gactctaatg cagctttagt tacaaaatca ttggttgaaa acttcaaaac tggaaacgtt
      421 gttgccgcgc taacatcatg gacacatact gtgactttat ggggcattga ggtagatgaa
      481 aaaactggca aaattaaaaa aggttatatc agtgactctg tagcagatca ggcaggaaac
      541 cttaaaatgg ttgaggtgac aggggattat gtacttgata ataaaggtaa tgaaattttt
      601 cgttttcttt atagttacta tgttccaaat ggaaataaat atgtcaccga tgtgtacgat
      661 atttacagca ttacttatgt gagtattgat gaaacgcgaa ataatggtac ttataaagac
      721 accactaacc gagaggattg taaattaagt ttagctccgg gagctgatgc tagttattgt
      781 ggtattagta acagtacaac aacgactact gcaactgaaa ataatagttc aaccaataca
      841 gagacagtag ctgagaatac taacgaagcg agtacagtca cgacagaacc agctccagaa
      901 gaaacagacg tagatgctca atcatcaaat acagagacat cggaaacaga gactgtaagt
      961 gaagtccctc caacaaaaga agctgttgca gaaaataacg agcaaatcgc tacatcaaac
     1021 actgaaacca cagtgtcaga agaaaatagc agtgaaatca cttcaacaga cagccctgcg
     1081 tcaaatgcag aaactgtagt gctagaaacg gaaaatacac caatcacgcc aactaatgga
     1141 aatagtagcg agccaagtgt agataatact gctcagtcag cagtagaaat gccagttgtt
     1201 gttactgaag aagataaacc aacagagtca gtggttgcag aaaataatga aaaaccgacc
     1261 gctacatcaa acactgaaac cacagtgtca gaagaaaata gcagtgaaat cacttcaaca
     1321 gacagctctg cgtcaaatgc agaaaccgta gtgctagaaa cggaaaatac accaagcact
     1381 ccagctaatg aaaatagcga tgagccaagt gtagataata ctgctcagcc aacagtagaa
     1441 acgccagttg ttgctactga agatgctaag ccaacagcag aagcagttgc agaaacaagc
     1501 gagcaaccga ccgctgtgcc aactcctgaa gttgcagagc cagcacctga gcctactcca
     1561 agcctagctt caactgaaac agcgactgta agcgaggtta aaccaacaga accagaaacc
     1621 gcagaacgtc tagcggtgaa atcattaaaa gaagctattg ctaattatca agagctaagt
     1681 tttgtaacca ctcgtgtcgg tgatgtaact cgttacctaa ttcaaagaga aggtattgat
     1741 attgctttaa ttgatccaaa tagcaagcag attttggtga acaatacggg cttggtgaac
     1801 tatgatttag ctcaagacca aaatggaaat atccatatca cgaaa
//
```

LOCUS MK617356 1371 bp DNA linear
BCT 23-SEP-2019
DEFINITION Glaesserella parasuis strain CF7066 espP2 gene, partial cds.
ACCESSION MK617356
VERSION MK617356
KEYWORDS .
SOURCE Glaesserella parasuis
ORGANISM Glaesserella parasuis
Bacteria; Proteobacteria; Gammaproteobacteria; Pasteurellales;
Pasteurellaceae; Glaesserella.

REFERENCE 1 (bases 1 to 1371)
AUTHORS Xu,X.
TITLE Direct Submission
JOURNAL Submitted (11-MAR-2019) Veterinary Infectious Diseases, College of
Veterinary Medicine, Huazhong Agricultural University, Shizishan
Street 1#, Wuhan, Hubei Province 430070, China
FEATURES Location/Qualifiers
source 1..1371
/organism="Glaesserella parasuis"
/mol_type="genomic DNA"
/strain="CF7066"
/serotype="5"
/isolation_source="Glasser's disease outbreak farm"
/db_xref="taxon:738"
/country="China"
/PCR_primers="fwd_name: espp2-f, fwd_seq:
cagacttattgggcaagtg, rev_name: espp2-r, rev_seq:
tttcgtgatatggatatttcca"
CDS <1..>1371
/note="putative extracellular serine protease
(autotransporter)"
/codon_start=1
/transl_table=11
/product="espP2"
/protein_id="QES71297"

/translation="QTYWASGVNQNSGWTADLQYPNQCWGAVAGNTLGWWKSRVKAQV

EFNEDTPKDSKAISGWIYKTYPHIQGGLQPYRGMEYFFSRFASGVKLYEEHNKKTYYE

SERGPTKISGGPFWTERYDSDAKLVTKSLIDNFKTGNVVAALTSQHHTVTLWGIEVDG

DGKIKKGWISDSVKDKAGNLKMVEVVGNYAKDNKGNDIFRFLYSYAVDGGRMYVTDLY

DIYSITYLSIDEARNNGTYKDTSNREDCRLSLAPGVSGSFCSINSTATNTTKVENVST

TNNNSTNLPTKVENSGYSNQVINAASASDDTVSSASENNDSVNTPENIENSNGVDIGN

NSTVSSANVENSSNEVSVINTPNTSSLPNDQGTKKIIVDNSESNAKDPIISKATESLT

KEINSYPELSFITKNMGDVTFYIVQKDGVDIALINPKTEQSLVSNKGLVIYDFKQDQN
GNIHITK"

ORIGIN

```
        1 cagacttatt gggcaagtgg agtcaatcaa aatagtggat ggactgctga tttacaatat
       61 ccaaatcagt gttggggtgc tgttgcagga aatactctcg gatggtggaa aagtagagta
      121 aaagcgcaag tagaatttaa cgaggatact cctaaagata gcaaagccat ttcaggatgg
      181 atatataaaa cttatccgca tatacagggt ggattacaac catatcgagg tatggagtat
      241 tttttctctc gttttgcatc aggagtaaaa ttatacgaag aacataataa aaaaacgtat
      301 tatgaaagtg agagaggtcc aacaaaaatt agtggtggtc cattctggac ggaacgttat
      361 gactcggatg ccaaattagt cactaaatct ttaattgata attttaagac aggaaacgtt
      421 gttgcagcac tcacttctca gcaccatacc gtaaccttat ggggaattga agttgatggg
      481 gatggtaaga taaaaaaagg atggattagt gattctgtta aggataaagc tggaaatctt
      541 aaaatggttg aggtggtagg aaattacgca aaagacaata aaggaaatga tatattccgt
      601 tttctttaca gctatgccgt tgacggtggt cgcatgtatg taacggatct ttatgatatt
      661 tatagtatta cctacctcag tatagatgag gcaagaaata atggtactta taaagataca
      721 tctaatcgtg aggattgtag gttaagtttg gctcctggtg tgtcaggaag tttttgttca
      781 attaatagta cagcaactaa tactacaaag gtagagaatg tatctactac taacaataac
      841 tcaacaaatc ttccaacaaa agttgaaaat agtggctatt caaatcaagt cattaatgct
      901 gcatcggcat cagatgatac tgtaagttca gcatccgaaa ataatgattc ggtaaataca
      961 ccggaaaata tcgaaaacag taatggggta gatataggta ataattcgac agtatcttcg
     1021 gcaaatgttg aaaatagtag taatgaggtt agtgtaatta atacaccaaa tacatcaagt
     1081 ctacctaacg atcaaggaac taaaaagatt attgttgata attcagaaag caacgcaaaa
     1141 gatcctatca taagcaaagc gacagaatct ttaacaaagg aaattaattc gtacccagaa
     1201 ctaagtttta tcactaagaa tatgggagat gtaacatttt atattgtgca aaaagatggt
     1261 gttgacatag ctttgattaa tccaaaaact gaacagtcgt tagttagtaa taagggatta
     1321 gtaatctatg atttcaaaca agaccaaaat ggaaatatcc atatcacgaa a
//
```
